# T cell immunoglobulin and mucin domain-3 is associated with disease activity and progressive joint damage in rheumatoid arthritis patients

**DOI:** 10.1097/MD.0000000000022892

**Published:** 2020-10-30

**Authors:** Haruki Matsumoto, Yuya Fujita, Tomoyuki Asano, Naoki Matsuoka, Jumpei Temmoku, Shuzo Sato, Makiko Yashiro-Furuya, Hiroshi Watanabe, Kiyoshi Migita

**Affiliations:** Department of Rheumatology, Fukushima Medical University School of Medicine, 1 Hikarigaoka, Fukushima, Fukushima, Japan.

**Keywords:** anti-citrullinated peptide antibody, matrix metalloproteinase-3, rheumatoid arthritis, T cell immunoglobulin and mucin domain-3

## Abstract

T cell immunoglobulin and mucin domain-3 (TIM-3) is a surface molecule expressed on immune cells which play a role in immune regulation. The aims of the present study were to determine whether circulating soluble T cell immunoglobulin domain and mucin-3 (sTIM-3) are elevated in rheumatoid arthritis (RA) patients, and investigate the relationships between sTIM-3 and clinical features of RA.

The study included 116 patients with established RA and 27 healthy control subjects. Serum levels of sTIM-3 were measured via the enzyme-linked immunosorbent assays (ELISA). Correlations between serum sTIM-3 and a range of parameters including anti-citrullinated peptide antibody (ACPA) titer, erythrocyte sedimentation rate (ESR), and matrix metalloproteinase-3 (MMP-3) were assessed.

Serum sTIM-3 was significantly elevated in RA patients compared with those in healthy subjects, and it was positively correlated with ACPA titer (*r* = 0.27 *P* = .005), ESR (*r* = 0.27, *P* = .004) and MMP-3 (*r* = 0.35, *P* < .001). In RA patients with high ACPA titers (≥200 U/mL), sTIM-3 was not correlated with ESR or MMP-3. Whereas, sTIM-3 was significantly correlated with ESR and MMP-3 in RA patients with low ACPA titers (<200 U/mL).

Serum sTIM-3 was increased in RA patients, and it was associated with proinflammatory markers and disease activity in RA patients under a particular ACPA status. Our data suggest that circulating sTIM-3 may be a useful biomarker for the determination of disease activity in RA patients.

## 1 Introduction

Rheumatoid arthritis (RA) is a chronic systemic autoimmune disease characterized by synovitis and subsequent inflammatory bone destruction.^[1]^ Previous studies indicate that imbalance between innate and adaptive immune systems can lead to excessive immune responses in rheumatoid synovium.^[2]^ Coinhibitory receptors such as cytotoxic T-lymphocyte-associated protein 4 have an important role in the regulation of T cell responses^[3]^ and are evidently effective targets in RA, in which coinhibitory T cell receptor expression can dampen the effector T cell responses.^[4]^

The “next wave” of coinhibitory receptor targets are currently being explored in chronic diseases.^[5]^ T cell immunoglobulin and mucin domain-3 (TIM-3) is a cell surface molecule that is expressed by CD4^+^ T helper 1 (Th1) cells.^[6]^ Functional studies investigating TIM-3 suggest that it is a negative regulator of Th1 immune responses.^[7]^ In support of this, anti-TIM-3 antibody was shown to exacerbate experimental autoimmune diseases such as experimental autoimmune encephalomyelitis.^[8]^ The C-type lectin galectin-9 was subsequently identified as a TIM-3 ligand.^[9]^ Galectin-9-mediated ligation of TIM-3 has been shown to induce cell death in TIM-3-expressing Th1 cells, leading to the amelioration of autoimmune diseases.^[10]^ TIM-3 was originally detected in adaptive immune cells, but following study demonstrated that TIM-3 is also expressed in innate immune cells.^[11]^ Expression of TIM-3 was detected in osteoclasts and its mononuclear precursor cells in rheumatoid synovium, and the TIM-3/galectin-9 regulatory system controls osteoclastogenesis and inflammatory bone destruction in RA.^[12]^ These findings suggest that rheumatoid inflammatory bone destruction may be influenced by upregulation of the TIM-3/galectin-9 axis. TIM-3 can be shed from the cell surface via a disintegrin and metalloproteinase with thrombospondin motifs 10-mediated or ADAM 17-mediated cleavage at the TIM-3 stalk region, resulting in a soluble form of T cell immunoglobulin domain and mucin-3 (sTIM-3) that can be detected in serum.^[13]^ We hypothesized that the TIM-3 axis is dysregulated in RA, and may be involved in rheumatoid inflammatory processes. In the current study serum levels of sTIM-3 were investigated in RA patients, and associations between sTIM-3 and clinical RA parameters were analyzed.

## 2 Methods

### 2.1 Patients

This observational single-center study included 116 consecutive RA patients. Patients were enrolled between February 2012 and September 2019, with follow-up ending in September 2019. We retrospectively reviewed the records of these RA patients. All patients were treated in Department of Rheumatology, Fukushima Medical School. All the patients met the 2010ACR/EULAR classification criteria for the disease.^[14]^ Probable RA or overlap syndromes were excluded.

The following clinico-demographic data were collected from the Medical Records Unit at Fukushima University Hospital: age, age at onset of RA, gender, disease activity score-28 for rheumatoid arthritis with erythrocyte sedimentation rate (ESR) (DAS28-ESR) score.^[15]^ As controls, 27 healthy subjects (11 males, 16 females, median age 40 years, interquartile range [IQR]; 35–49 years) were included. This study was conducted in accordance with the principles of the Declaration of Helsinki. Ethical approval for this study (No. 2019097) was provided by the Ethics Committee of Fukushima Medical University.

### 2.2 Measurement of clinical disease activity

All patients were analyzed for the clinical assessment at baseline, including 28-joint swollen and tender joint counts (28-SJC and 28-TJC, respectively), physician and patient global assessment with visual analogue scales (0–100 mm) and ESR (mm). The composite disease activity indices were subsequently calculated: DAS28-ESR.^[15]^ Result of this score was reported in quantitative value divided into 4 categories: remission with score of <2.6, mild activity if score of ≥2.6 to <3.2, moderate activity if score of ≥3.2 to <5.1, and high activity if score of ≥5.1. The patients’ anti-CCP antibodies were analyzed using commercially available second-generation chemiluminescent enzyme immunoassay kits (STACIA MEBLux CCP test, Medical and Biological Laboratories, Aichi, Japan) according to the manufacturer's instructions. Radiographs were taken of both hands of each patient. Two rheumatologists, blinded to the patient's identify and functional status, independently graded each hand radiographs and assigned as Steinbrocker radiographic stage.^[16]^

### 2.3 Enzyme-linked immunosorbent assays (ELISA) methods

Serum concentrations of sTIM-3were measured using enzyme-linked immunosorbent assay kit (R&D Systems, Minneapolis, MN) according to the manufacturer's instruction.

### 2.4 Statistical analysis

Results were non-normally distributed and are presented throughout the manuscript with median and IQR, and were compared by the Mann-Whitney *U* test. Correlations between continuous variables were analyzed by the Spearman's rank correlation test. All data entry and statistical analyses were performed using SPSS Statistics version 22.0 (IBM, Armonk, NY). In all the analyses, a 2-tailed *P* < .05 was considered statistically significant.

## 3 Results

### 3.1 Demographic data of enrolled RA patient

The study included 116 RA patients and 27 healthy controls. The demographics and clinical characteristics of the RA patients are shown in Table 1. Among 116 patients with RA, 83 (71.6%) were female and their mean age was 63.5 years. The majority of the RA patients were taking disease-modifying anti-rheumatic drugs (DMARDs), mostly methotrexate (59/116 50.9%), and biologics (38/116 32.8%).

**Table 1:**
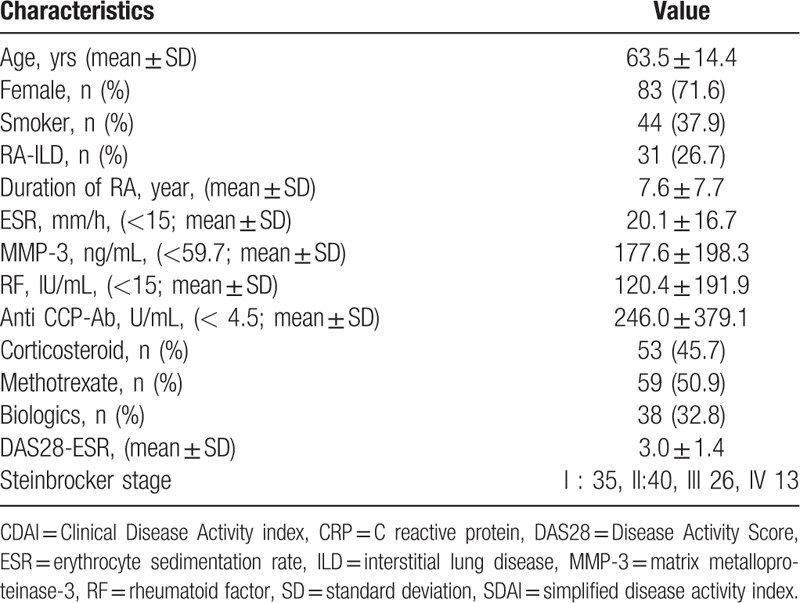
Baseline demographics, clinical characteristics, and profiles (n = 116).

### 3.2 Serum sTIM-3 concentrations in RA patients

**Figure 1:**
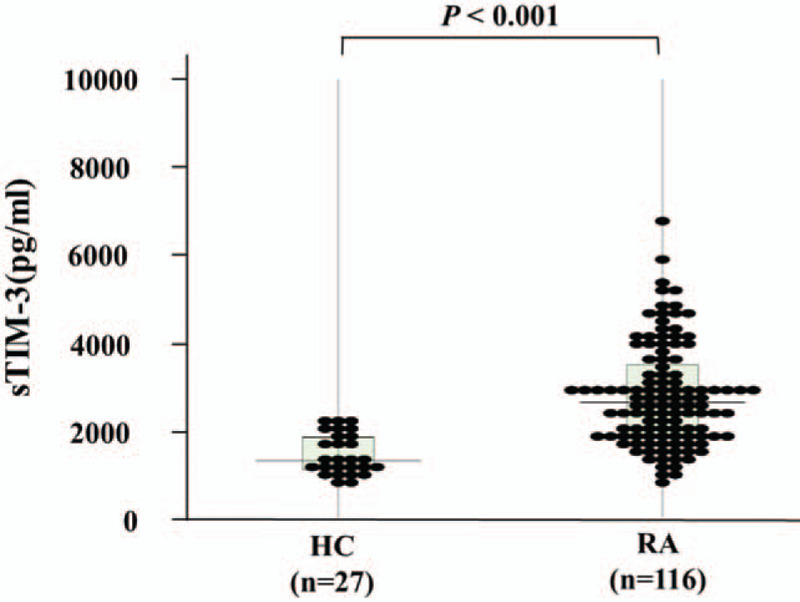
Serum levels of sTIM-3 in RA patients and controls. Serum levels of sTIM-3 in RA patients (n = 116) were significantly higher compared to those in healthy subjects (n = 27). Results were presented with median and were compared by the Mann-Whitney *U* test. RA = rheumatoid arthritis, sTIM-3 = soluble T cell immunoglobulin domain and mucin-3.

Serum sTIM-3 levels were significantly higher in RA patients compared to those in the healthy subjects (Fig. 1). There were significant positive correlations between serum sTIM-3 and rheumatoid inflammatory markers such as ESR (*r* = 0.27, *P* = .004), matrix metalloproteinase-3 (MMP-3, *r* = 0.35, *P* < .001), and anti-citrullinated peptide antibody (ACPA) titers (*r* = 0.27 *P* = .005). There was no significant difference in serum levels of sTIM-3 (median: 2819 pg/ml vs 2615 pg/ml, *P* = .26), titers of ACPA (median: 56.4 U/ml vs 60.1 U/ml, *P* = .82) and titers of RF (median: 64.0 IU/ml vs 30.0 IU/ml, *P* = .69) between RA patients treated with and without biologics.

### 3.3 Relationships between sTIM-3 and ACPA titers

**Figure 2:**
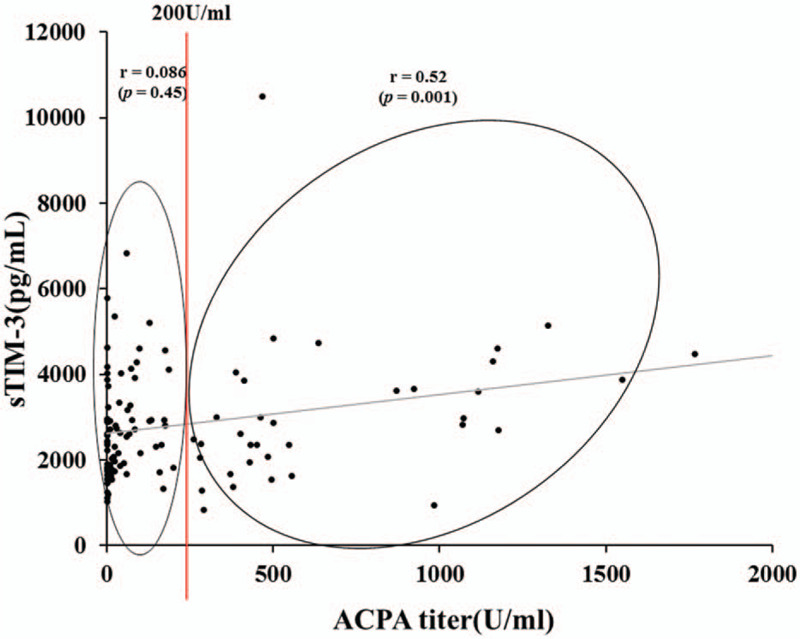
Relationship between ACPA titers and serum levels of sTIM-3 in patients with RA. Levels of ACPA titers were measured and correlation analysis with serum levels of sTIM-3 was performed. There was no significant correlation between serum levels of sTIM-3 and ACPA titers in RA patients with low titers of ACPA (<200 U/ml). Whereas there was a significant modest correlation between serum levels of sTIM-3 and ACPA titers in RA patients with high titers of ACPA (≧200 U/ml). ACPA = anti-citrullinated peptide antibody, RA = rheumatoid arthritis, sTIM-3 = soluble T cell immunoglobulin domain and mucin-3.

**Figure 3:**
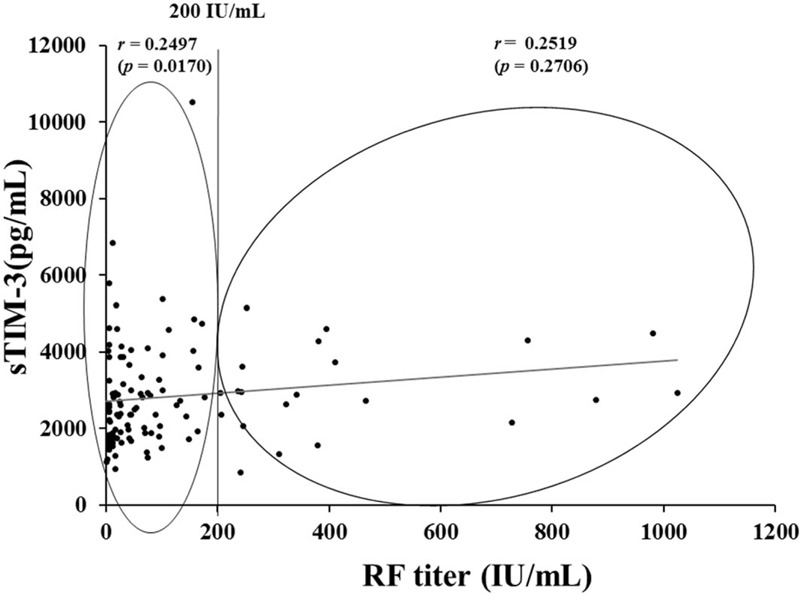
Relationship between RF and serum levels of sTIM-3 in patients with RA. Levels of RF were measured and correlation analysis with serum levels of sTIM-3 was performed. There was a weak correlation between sTIM-3 an RF (*r* = 0.26, *P* = .006), in total RA patients. Whereas there was no significant correlation between serum levels of sTIM-3 and RF titers in RA patients with high titers of RF (≧200 IU/ml). RA = rheumatoid arthritis, RF = rheumatoid factor, sTIM-3 = soluble T cell immunoglobulin domain and mucin-3.

To further evaluate the capacity of serum sTIM-3 to differentiate between RA phenotypes, distribution patterns of serum sTIM-3 values were analyzed in conjunction with ACPA titers. In a two-dimensional map consisting of serum sTIM-3 and ACPA titer, 2 distinct groups were apparent (Fig. 2). The cutoff value of ACPA titer (200 U/ml) was determined according to the ability to extract the strongest correlation between sTIM-3 and ACPA titer. When RA patients were grouped according to the presence of high ACPA titers (≧200 U/ml), some correlations between circulating sTIM-3 and clinical features were identified. There was a significant modest correlation between sTIM-3 and ACPA titer in RA patients with high ACPA titers (≥200 U/mL *r* = 0.508, *P* = .002). Whereas, there was no correlation between sTIM-3 and ACPA titer in RA patients with low ACPA titers (<200 U/mL). There were differential correlations between serum sTIM-3 levels and ACPA titers in these 2 groups, suggesting that sTIM-3 could be modulated by ACPA titers. We also evaluated the correlations between sTIM-3 and RF (Fig. 3). There was a weak correlation between sTIM-3 an RF (*r* = 0.26, *P* = .006) in total RA patients; however, there was no significant correlation between sTIM-3 and RF in RA patients with high titers of RF.

### 3.4 Relationships between sTIM-3 and clinical and laboratory parameters (Fig. 4)

In RA patients with low ACPA titers (<200 U/mL), serum sTIM-3 was significantly correlated with the inflammatory markers, ESR (*r* = 0.36, *P* = .001) and MMP-3 (*r* = 0.38, *P* < .001). Whereas corresponding correlations were not significant in RA patients with high ACPA titers (≥200 U/mL). These findings suggest that serum sTIM-3 upregulation may be associated with autoimmune responses in RA patients with high ACPA titers, whereas serum sTIM-3 was upregulated in response to inflammatory mediators in RA patients with low ACPA titers.

**Figure 4:**
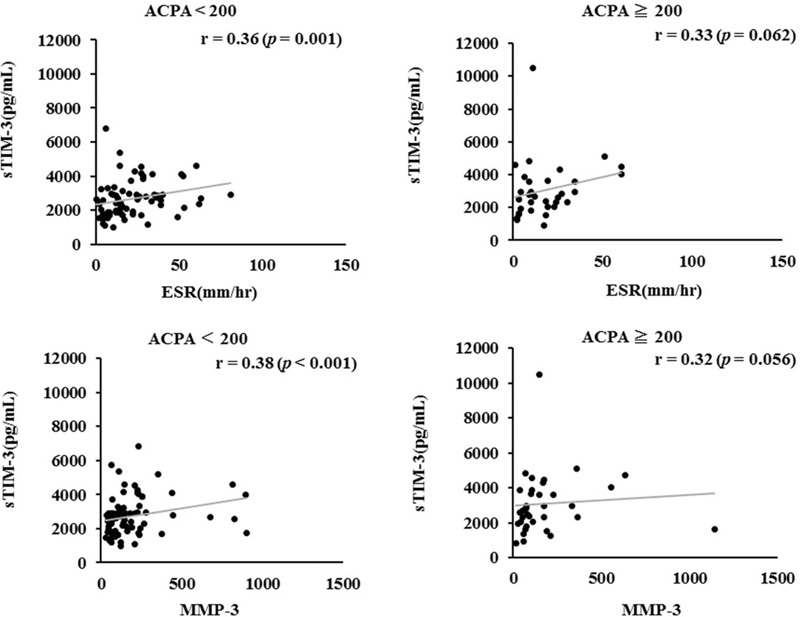
Correlation between serum levels of sTIM-3 and proinflammatory markers (A: ESR, B: MMP-3) in the sub-grouped RA patients according to the titers of ACPA. Correlation analysis of serum levels of sTIM-3 and clinical parameters (A: ESR, B: MMP-3) does not show a relationship in RA patients with high titers of ACPA (≧200U/ml). Whereas there was a significant positive correlation between clinical parameters (A: ESR, B: MMP-3) in RA patients with low titers of ACPA (<200 U/ml). ACPA = anti-citrullinated peptide antibody, MMP-3 = matrix metalloproteinase-3, RA = rheumatoid arthritis, sTIM-3 = soluble T cell immunoglobulin domain and mucin-3.

In RA patients with low ACPA titers (<200 U/mL), circulating sTIM-3 was significantly higher in C reactive protein (CRP)-positive patients than it was in CRP-negative patients (2871 pg/mL, [IQR 2145–3921] vs 2081 pg/mL, [IQR 1714–2840], *P* = .007). Conversely, in patients with high ACPA titers (≥200 U/mL) there was no significant difference in circulating sTIM-3 between CRP-positive and CRP-negative patients (2998 pg/mL, [IQR 2640–3960] vs 2385s pg/mL, [IQR 1712–3826], *P* = .20).

### 3.5 Relationships between sTIM-3 and RA clinical outcomes

**Figure 5:**
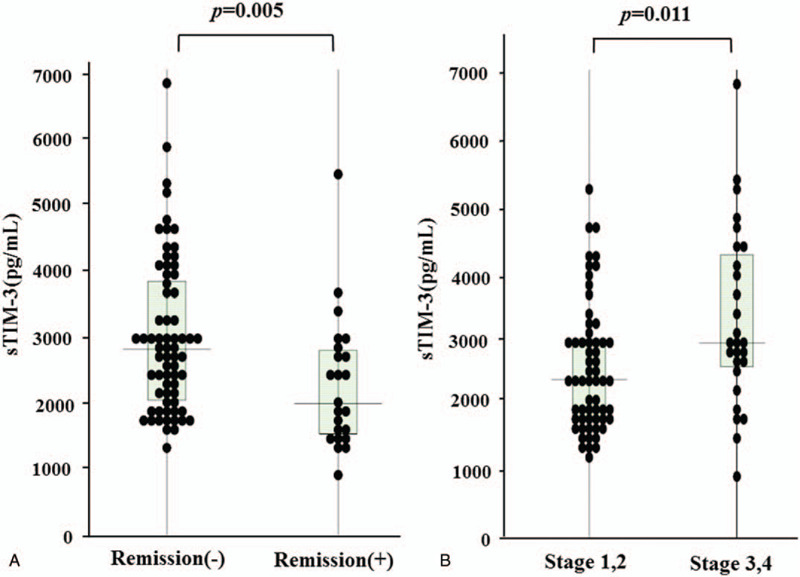
Serum levels of sTIM-3 were compared between RA patients with or without DAS28-ESR clinical remission (A) or those with or without advanced joint damage (B). A; Serum sTIM-3 were significantly lower in RA patients with CR compared to those without CR. CR = clinical remission. B: Serum sTIM-3 were significantly higher in RA patients with advance joint damage (stage III or IV) compared to those without advance joint damage. RA = rheumatoid arthritis, sTIM-3 = soluble T cell immunoglobulin domain and mucin-3.

**Figure 6:**
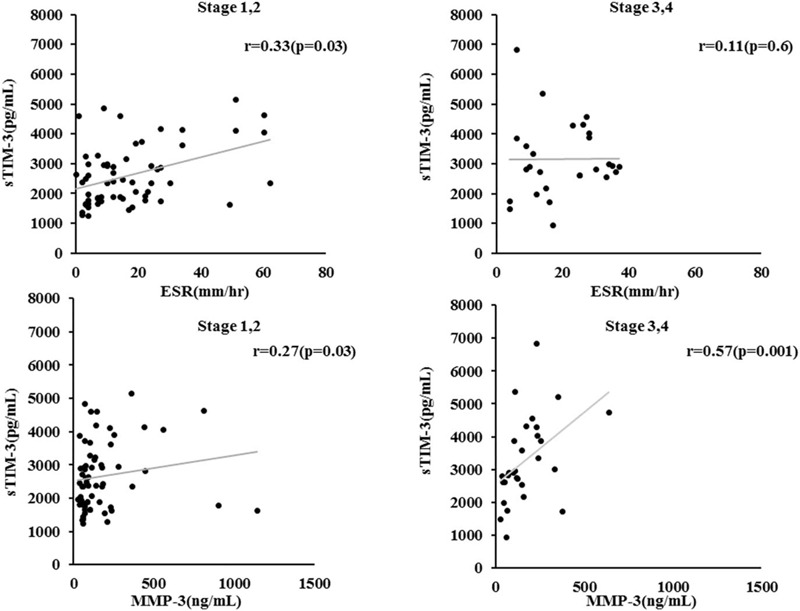
Correlation between serum levels of sTIM-3 and proinflammatory markers (A: ESR, B: MMP-3) in the sub-grouped RA patients according to the advanced joint damage. Serum sTIM-3 significantly correlated with ESR not with MMP-3 in RA patients without the advanced joint damage (stage III or IV). Whereas there was a significant positive correlation between sTIM-3 and MMP-3, not with ESR in RA patients with the advanced joint damage. ESR = erythrocyte sedimentation rate, MMP-3 = matrix metalloproteinase-3, RA = rheumatoid arthritis.

From a clinical point of view, the circulating sTIM-3 was compared according to the presence or absence of clinical remission in RA patients (Fig. 5A). Serum sTIM-3 was significantly higher in RA patients without clinical remission compared to those with clinical remission (2852 pg/ml [IQR: 2013–3584] vs 1954 pg/ml [IQR: 1611–2875], *P* = .005). As shown in Figure 5B, RA patients with advanced joint damage (stage III or IV) had significantly higher levels of serum sTIM-3 than those without advanced joint damage (2830 pg/ml [IQR: 2181–4047] vs 2428 pg/ml [IQR: 1927–3175], *P* = .011). There was a differential relationship between circulating sTIM-3 and rheumatoid inflammatory markers in the presence or absence of advanced joint damage (stage III or IV). In RA patients with advanced joint damage (stage III or IV), serum sTIM-3 significantly correlated with MMP-3 but not with ESR. Conversely, in RA patients without advanced joint damage, circulating sTIM-3 was significantly correlated with ESR, whereas there was a weak correlation between sTIM-3 and MMP-3 (Fig. 6).

## 4 Discussion

RA is the most common autoimmune disease characterized by immune-mediated etiology associated with synovial inflammation and articular destruction.^[17]^ Genetic and environmental factors play a role in RA risk, and complex interactions between multiple cytokines, immune cells, and biological pathways contribute to RA development.^[18]^ Intricate relationships between genetic, environmental, and cellular factors may contribute to the heterogeneity of RA.^[19]^ Few clinically useful biomarkers exist for RA in terms of disease phenotype, treatment response, and disease progression. ACPA production tends to be associated with a more aggressive course,^[20]^ however, other prognostic markers could be involved in RA phenotypes.^[21]^ New biomarkers for RA disease phenotypes, joint outcomes, and severity may be useful to predict the therapeutic outcomes in RA patients. Various RA-related biomarkers have been identified, but which of these are the most informative with regard to immunomodulatory factors and RA phenotypes remains to be established.^[22]^

A growing body of evidence suggested the contributions of coinhibitory receptors, including TIM-3, to the immune-mediated disorders.^[23]^ Our proposed hypothesis is that the TIM-3/galectin-9 axis is implicated in maintaining the regulation of adaptive and innate immune responses in RA. In the current study we investigated serum levels of sTIM-3 in patients with established RA. Circulating sTIM-3 was significantly elevated in RA patients and it was correlated with ACPA titers and rheumatoid inflammatory markers, reflecting the activation of adaptive and innate immunity. In RA patients with low ACPA titers, there were positive correlations between sTIM-3 and rheumatoid inflammatory markers. Whereas neither of these parameters was correlated with sTIM-3 in RA patients with high ACPA titers. These findings suggest that circulating sTIM-3 levels are differentially regulated in RA patients depending on their ACPA status.

TIM-3 is a surface molecule expressed on immune cells that plays an important role in immune regulation.^[9]^ Identification of galectin-9 as a TIM-3 ligand has established the TIM-3/galectin-9 pathway as a regulator of Th1 immunity.^[7]^ Ligation of TIM-3 by galectin-9 in a mouse model of collagen-induced arthritis effectively inhibited the severity of the condition, including inflammatory cell infiltration and bone distinction.^[10]^ Conversely, blocking the TIM-3/galectin-9 pathway via a monoclonal antibody or gene knockout can exacerbate the autoimmune conditions.^[24]^ High expressions of TIM-3 in synovial tissues and peripheral mononuclear cells have been demonstrated in RA patients.^[25]^ TIM-3 was first described as an inhibitory receptor on T cells, it is now known to be expressed on innate immune cells.^[26]^ TIM-3 expressed on innate immune cells is presumed to activate co-inhibitory functions^[27]^; however, the mechanisms by which TIM-3 regulates innate immunity are unclear. Although galectin-9 is proposed as a ligand for TIM-3, TIM-3 function was not exclusively restricted by galectin-9.^[5]^ Circulating TIM-3 may reflect the expression of membrane-bound form of TIM-3 and the activation status of TIM-3 pathway under inflammatory conditions,^[28]^ since the positive correlation between TIM-3 expression and inflammatory cytokines was demonstrated.^[29]^ However, the underlying mechanisms by which TIM-3 affects the rheumatoid inflammatory process, remains unclear.

More recently, we and other group demonstrated the elevated serum levels of Galectin-9 in RA patients.^[30,31]^ Furthermore, beneficial effect of galectin-9 on RA through the induction of apoptosis of synovial fibroblasts had been suggested.^[32]^ It can be concluded that the TIM-3/galectin-9 pathway is activated in RA as an anti-immune mediators. However, this pathway can be modulated by the soluble form of TIM-3 which is shedded form TIM-3 expressing immune cells.^[13,33]^ TIM-3/galectin-9 interaction may result in T cell exhaustion, on the contrary, sTIM-3 seems to have alternative effects against this feedback mechanism. Given the apparent complexity of the function of TIM-3, further studies are needed to determine the source of serum sTIM-3 and its role in rheumatoid inflammatory progression in RA patients.

Predicting drug responses and clinical courses in RA patients is challenging due to the heterogeneity of individual clinical phenotypes. ACPA-positive and ACPA-negative patients may exhibit similar clinical manifestations at baseline, but different phenotypes with respect to subsequent radiographic progression.^[34,35]^ Interestingly, in the present study there was a modest correlation between sTIM-3 concentration and MMP-3 in RA patients with advance joint damage (stage III or IV). RA is characterized by 2 key events, immune activation and subsequent inflammatory bone damage.^[35]^ MMP-3 is mainly produced by rheumatoid synovial fibroblasts or osteoclasts activated by inflammatory cytokines, and it can degrade components of the cartilage extracellular matrix (ECM), leading to rheumatoid bone destruction.^[36]^ Therefore, the modest correlation between sTIM-3 and MMP-3 in RA patients with advanced bone damage suggest that sTIM-3 upregulation may reflect the activated status of rheumatoid stromal cells, including MMP-3 induction, under the rheumatoid bone destruction processes.

There are several potential limitations of this study that should be considered. First, the patient population was relatively small and a larger study is essential to confirm our results. Second, all patients with RA and healthy individuals in this study were Japanese, additional studies in other ethnic groups are needed to verify these findings. Third, it will be important to examine the longitudinal changes of serum sTIM-3 levels in patients with RA and to assess their clinical course in the future studies.

## 5 Conclusions

Circulating sTIM-3 was significantly higher in RA patients without clinical remission compared with those with clinical remission as well as healthy subjects. Circulating sTIM-3 significantly correlated with rheumatoid inflammatory markers or matrix-degrading markers in RA patients under particular ACPA status, or degrees of joint damage. Combined consideration of sTIM-3 and ACPA titers may facilitate distinctions between degrees of RA disease activity.

## Acknowledgments

We are grateful to Ms Sachiyo Kanno for her technical assistance in this study.

## Author contributions

**Conceptualization:** Haruki Matsumoto, Yuya Fujita, Tomoyuki Asano, Naoki Matsuoka, Jumpei Temmoku, Shuzo Sato, Makiko Yashiro-Furuya, Hiroshi Watanabe.

**Data curation:** Haruki Matsumoto, Yuya Fujita, Tomoyuki Asano, Naoki Matsuoka, Jumpei Temmoku, Shuzo Sato, Makiko Yashiro-Furuya, Hiroshi Watanabe.

**Formal analysis:** Haruki Matsumoto, Kiyoshi Migita.

**Funding acquisition:** Kiyoshi Migita.

**Investigation:** Kiyoshi Migita.

**Methodology:** Yuya Fujita, Kiyoshi Migita.

**Project administration:** Kiyoshi Migita.

**Resources:** Kiyoshi Migita.

**Software:** Kiyoshi Migita.

**Supervision:** Kiyoshi Migita.

**Validation:** Kiyoshi Migita.

**Visualization:** Kiyoshi Migita.

**Writing – original draft:** Haruki Matsumoto, Kiyoshi Migita.

**Writing – review & editing:** Kiyoshi Migita.
